# Increased circulating vascular endothelial growth factor in acute myeloid leukemia patients: a systematic review and meta-analysis

**DOI:** 10.1186/s13643-020-01368-9

**Published:** 2020-05-06

**Authors:** Mingzhu Song, Huiping Wang, Qianling Ye

**Affiliations:** Department of Hematology, The Second Affiliated Hospital of Anhui Medical University, Hefei, 230601 Anhui People’s Republic of China

**Keywords:** Acute myeloid leukemia, Vascular endothelial growth factor, Meta-analysis

## Abstract

**Background:**

Vascular endothelial growth factor (VEGF) is one of the angiogenesis regulators, which plays an important role in tumor angiogenesis and tumor progression. Current studies have found that VEGF plays an important role in hematologic diseases including acute myeloid leukemia (AML). However, the circulating levels of VEGF in AML were still controversial among published studies.

**Methods:**

Three databases including PubMed, EMBASE, and Cochrane Library databases were searched up to February 2020. All articles included in the meta-analysis met our inclusion and exclusion criteria. Studies will be screened and data extracted by two independent investigators. The Newcastle-Ottawa Scale (NOS) and the Risk of Bias In Non-randomized Studies of Interventions (ROBINS-I) tool were applied to evaluate the quality of the included studies. A random-effects model was applied to pool the standardized mean difference (SMD). Heterogeneity test was performed by the *Q* statistic and quantified using *I*^2^. All statistical analysis was conducted in Stata 12.0 software.

**Results:**

Fourteen case-control studies were finally included in this systematic review and meta-analysis. Heterogeneity was high in our included studies (*I*^*2*^ = 91.1%, *P* < 0.001). Sensitivity analysis showed no significant change when any one study was excluded using random-effect methods (*P* > 0.05). Egger’s linear regression test showed that no publication bias existed (*P* > 0.05). Patients with AML, mainly those newly diagnosed and untreated, have higher VEGF levels (SMD = 0.85, 95% CI 0.28–1.42). Moreover, AML patients in *n* ≥ 40 group, plasma group, Asia and Africa group, and age ≥ 45 group had higher circulating VEGF levels (all *P* < 0.05).

**Conclusions:**

Compared to healthy controls, our meta-analysis shows a significantly higher level of circulating VEGF in AML patients, and it is associated with sample size, sample type, region, and age.

## Background

Acute myeloid leukemia (AML) is a heterogeneous hematopoietic malignancy, characterized by the accumulation of uncontrolled growth of hematopoietic progenitor cells in the bone marrow and peripheral blood [1]. AML is the most common type of acute leukemia in adults, which usually affects the elderly (> 65 years old), and the survival of elderly AML patients is very poor [2]. Studies have shown that the development of AML is closely related to the interactions between leukemic blasts and stromal cells in the bone marrow microenvironment [3]. Bone marrow biopsies in AML patients showed more endothelial cells than those who did not have malignancy. AML blasts can produce and secrete vascular endothelial growth factor (VEGF) [3, 4].

VEGF, also termed VEGF-A, is one of the most important positive mediators of physiological and pathological angiogenesis [5]. VEGF traditionally has been recognized as a paracrine factor in both developmental and pathological settings [6]. It promotes the processes of vascular growth and remodeling and provides endothelial cells with mitosis and survival stimulation [5]. It has been demonstrated to be closely related to the progression of various cancers and tumor angiogenesis in human [7]. Expression and activation of VEGF/VEGF receptors are necessary for normal hematopoietic function. The increased level of serum and intracellular VEGF is associated with the growth, diffusion, metastasis, and poor prognosis of solid tumors [8]. So far, studies have focused mainly on various solid tumors. For example, it has been shown that the level of VEGF is overexpressed in head and neck cancer [9]. What is more, several meta-analyses have shown that high VEGF expression is associated with poorer overall survival in patients with osteosarcoma, oral cancer, and gastric cancer [10–12].

In hematologic malignancies, VEGF stimulates mitotic responses; triggers growth, survival, and migration; and upgrades the self-renewal of leukemia progenitor cells [13]. Increased levels of VEGF have been observed in a variety of hematologic malignancies, such as multiple myeloma (MM), non-Hodgkin lymphoma (NHL), chronic myeloid leukemia (CML), chronic lymphocytic leukemia (CLL), chronic myelomonocytic leukemia (CMML), myelodysplastic syndromes (MDS), and acute myeloid leukemia (AML) [14–17]. AML blasts can enhance autocrine VEGF signaling, and thereby regulating the angiogenesis induced by paracrine vascular endothelial cells and promoting the progression of AML [18]. However, the level of VEGF in AML patients remains controversial. One study showed that total serum VEGF in AML patients was significantly lower than that in healthy controls, possibly due to thrombocytopenia in AML patients [19]. Several studies have shown higher levels of VEGF in AML patients than healthy controls [20–24]. Besides, Aref et al. [25, 26], Aguayo et al. [16, 27], and Wang et al. [28, 29] all showed elevated level of VEGF in AML patients compared to normal control. Wierzbowska et al. [30] and Dincaslan et al. [31] showed different results; they showed that there was no significant difference between the AML patients and healthy controls. We conducted a meta-analysis of the topic to further clarify the results.

## Methods

The protocol of this systematic review has not been registered with PROSPERO. This review is written in accordance with the Preferred Reporting Items for Systematic Reviews and Meta-Analyses (PRISMA) statement guideline [32]. A completed copy of the PRISMA checklist is provided in Additional file 1.

### Search strategy

Three databases including PubMed, EMBASE, and Cochrane library databases were searched. The following keywords were searched in all fields: “acute myeloid leukemia” OR ”AML” OR “acute nonlymphocytic leukemia” OR ”ANLL”, ”vascular endothelial growth factor” OR “Vasculotropin” OR ”VEGF” OR “VEGF-A”. No method or language restrictions were applied, and studies from all countries were eligible. No publication years restricted, and the search deadline was February 2020. The included literature was screened to meet the inclusion and exclusion criteria below. The detailed search strategy is available in Additional file 2.

### Inclusion criteria and exclusion criteria

Studies included should follow the inclusion criteria:

1. Included AML patients were newly diagnosed, relapsed, or secondary.
2. Detailed data about circulating VEGF levels in both AML patients and healthy controls were available.
3. The value of VEGF was derived from serum or plasma.

Exclusion criteria were as follows:

1. The value of VEGF in all AML patients and healthy control was derived from serum or plasma, excluding samples from bone marrow or cells.
2. No sufficient data for detailed analysis, conference abstracts, reviews, full-text unavailable, no healthy control, systematic review and meta-analysis, and articles from which the full text was not available.

**Fig. 1:**
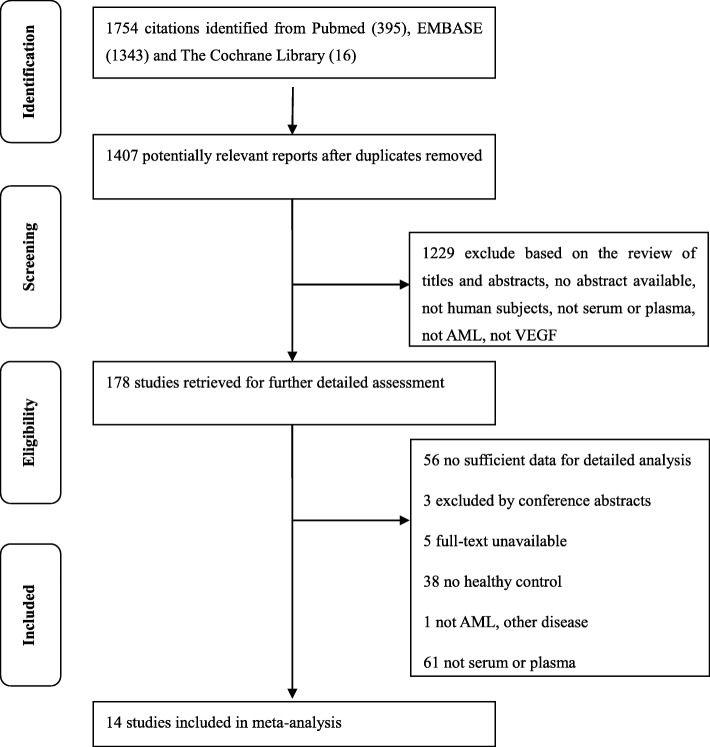
Flow chart of selected articles. After excluding inappropriate articles, 14 articles were included in the final analysis. AML: acute myeloid leukemia; VEGF: vascular endothelial growth factor

The specific literature inclusion and exclusion are shown in Fig. 1.

### Data extraction

Extract the following information from the articles that are included in the meta-analysis: first author’s name, year of publication, region, sample size, sample type, age, study type, assay method, and the mean and standard deviation of VEGF in both AML and healthy controls. Some articles provided standard error (SE), median, and min–max (ranges) values due to low sample volume in their original works, so we used some formulas to convert this data to mean and standard deviation [33–35]. The specific calculations are presented in Additional file 3. Two independent investigators (Mingzhu Song and Huiping Wang) used the Newcastle-Ottawa quality assessment scale (NOS) and the Risk Of Bias In Non-randomized Studies of Interventions (ROBINS-I) assessment tool (see Additional file 4) to evaluate the quality of the included studies [36, 37].

### Statistical analysis

The DerSimonian and Laird approach (DL) is the standard method of random-effects meta-analysis, and it was used in our meta-analysis [38]. The standardized mean difference (SMD) and its 95% confidence interval (95%CI) were described by a forest plot. A heterogeneity test based on *Q* statistic and *I*^*2*^ = [(*Q* − *df*)/*Q*] × 100% was carried out [39]. *I*^*2*^ was used for quantifying inconsistency: a value of 0% indicates that no heterogeneity was observed, and the larger the value, the stronger the heterogeneity. *I*^2^ values of 25%, 50%, and 75% were qualitatively classified as low, moderate, and high heterogeneity [40]. Funnel plot was used to visually evaluate publication bias, and Egger’s linear regression test was applied to assess asymmetry of the funnel plot [41]. Sensitivity analysis was applied to detect the stability of the results, and subgroup analysis was performed to evaluate the potential sources of heterogeneity. All data analyses were performed using Stata 12.0 software.

## Results

### Study characteristics

A total of 1754 potential articles were acquired from three major databases initially, and 347 articles were excluded due to duplicate publication. After screening of titles and abstracts, 178 studies were retrieved for further detailed assessment. Fourteen articles with 649 AML patients and 261 healthy controls were finally included in the meta-analysis according to the inclusion and exclusion criteria (Fig. 1). The basic characteristics of the selected studies are presented in Table 1.

**Table 1:** Characteristics of abstracted studies

Author, year	Region	Patients with AML				Control				Sample type	Assay method	Study type	Criteria for the classification of AML	NOS
		*N*	Age, mean ± sd, years	% female	VEGF mean ± sd, pg/ml	*N*	Age, mean ± sd, years	% female	VEGF, mean ± sd, pg/ml					
Aguayo et al., 2000 [16]	American	115	NA	NA	30.43 ± 69.63	11	NA	NA	32.63 ± 9.50	Plasma	ELISA	Case-control	NA	6
Aguayo et al., 2002 [27]	American	58	NA	NA	30.63 ± 92.09	43	39.00 ± 13.75	NA	27.30 ± 17.08	Plasma	ELISA	Case-control	FAB	7
Aref et al., 2002 [25]	Egypt	63	47.00 ± 12.50	28/63	78.00 ± 47.25^a^	15	NA	NA	33.03 ± 13.76^a^	Plasma	ELISA	Case-control	FAB	7
Wang et al., 2003 [28]	China	39	42.00 ± 14.75	19/39	135.30 ± 87.90	12	NA	NA	80.60 ± 33.10	Plasma	ELISA	Case-control	NA	6
Xie and Qi, 2003 [24]	China	25	NA	NA	201.43 ± 51.84	30	36.71 ± 11.75	14/30	100.53 ± 47.67	Serum	ELISA	Case-control	FAB	6
Wierzbowska et al., 2003 [30]	Poland	38	NA	NA	32.60 ± 651.20	12	NA	NA	44.40 ± 31.60	Plasma	ELISA	Case-control	FAB	6
Wang et al., 2004 [29]	China	107	42.00 ± 11.83	59/107	154.75 ± 109.98	26	NA	NA	99.91 ± 41.87	Plasma	ELISA	Case-control	FAB	6
Kim et al., 2005 [17]	Korea	28	41.50 ± 14.75^b^	NA	54.30 ± 113.15	17	NA	NA	238.95 ± 136.25	Serum	ELISA	Case-control	NA	6
Aref et al., 2005 [26]	Egypt	43	NA	NA	373.90 ± 222.95	10	NA	NA	138.00 ± 14.86	Plasma	ELISA	Case-control	FAB	6
Erdem et al., 2006 [23]	Turkey	15	32.60 ± 18.80	5/15	110.10 ± 120.90	20	34.00 ± 11.90	8/20	69.90 ± 24.40	Serum	ELISA	Case-control	NA	7
Zhao and Zhao, 2007 [22]	China	15	NA	NA	377.49 ± 146.31	15	NA	NA	77.11 ± 21.37	Serum	ELISA	Case-control	NA	6
Dincaslan et al., 2010 [31]	Turkey	7	7.17 ± 4.84	4/7	286.50 ± 328.81	20	NA	NA	190.50 ± 117.50	Serum	ELISA	Case-control	FAB	7
Song et al., 2015 [21]	China	28	NA	NA	74.97 ± 29.04	10	NA	NA	41.76 ± 10.03	Serum	ELISA	Case-control	FAB/WHO	7
Yang et al., 2016 [20]	China	68	51.00 ± 12.37	32/68	293.21 ± 57.54	20	49.00 ± 8.94	10/20	133.00 ± 24.65	Plasma	ELISA	Case-control	FAB/WHO	7

*N* number, *NA* not available, *VEGF* vascular endothelial growth factor, *AML* acute myeloid leukemia, *ELISA* enzyme-linked immunosorbent assay, *NOS* Newcastle-Ottawa Scale

^a^Nanograms per milliliter

^b^Of 30 people’s age (mean ± sd)

### Meta-analysis results

#### Heterogeneity test results

**Fig. 2:**
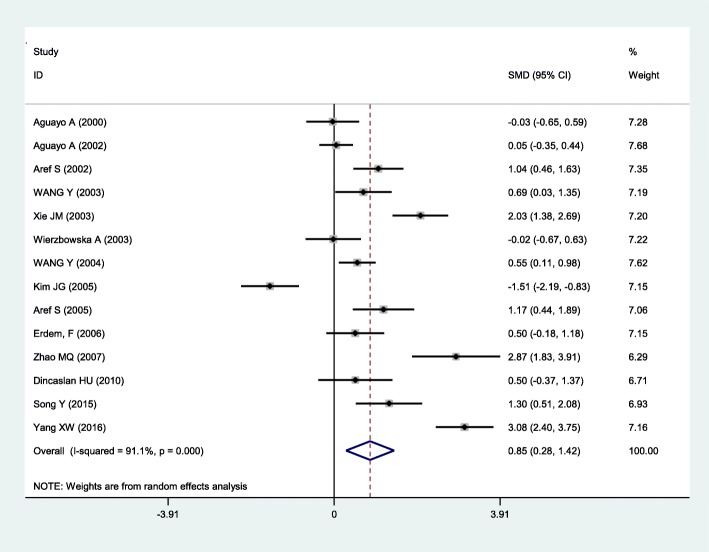
Meta-analysis of 14 studies reporting on VEGF in AML compared with controls. SMD: standardized mean difference

The result of heterogeneity test showed that there was significant heterogeneity across studies (*I*^*2*^ = 91.1%, *P* < 0.001) (Fig. 2), and the random-effects model was used for following data analyses. Random-effects model attempted to generalize findings beyond the included studies by assuming that the selected studies are random samples from a larger population [42].

#### Overall effects and subgroup analysis

AML patients had significantly higher levels of serum/plasma VEGF (*P* < 0.001, SMD = 0.85, 95% CI = 0.28 to 1.42, Fig. 2) when compared to healthy controls. Subgroup analyses showed that sample size ≥ 40 (SMD = 0.95, 95% CI = 0.14 to 1.77), plasma (SMD = 0.80, 95% CI = 0.16 to 1.44), Asia and Africa (SMD = 1.09, 95% CI = 0.39 to 1.80), and age ≥ 45 (SMD = 2.05, 95% CI = 0.06 to 4.04) had higher level of VEGF in AML (Table 2).

**Table 2:** Subgroup analysis of VEGF levels in AML

Stratification group	*N*	SMD (95% CI)	Heterogeneity test			Publication bias	
			*Q*	*P*	*I*^*2*^ (%)	t	*P*
Total	14	0.85 (0.28 to 1.42)	146.87	< 0.001	91.1	− 0.75	0.467
Sample size							
*n* ≥ 40	6	0.95 (0.14 to 1.77)	66.45	< 0.001	92.5	− 1.13	0.321
*n* < 40	8	0.77 (− 0.11 to 1.65)	80.21	< 0.001	91.3	− 0.92	0.392
Sample type							
Plasma	8	0.80 (0.16 to 1.44)	70.81	< 0.001	90.1	− 0.85	0.430
Serum	6	0.93 (− 0.28 to 2.14)	75.64	< 0.001	93.4	− 0.66	0.545
Region							
Asia and Africa	11	1.09 (0.39 to 1.80)	119.16	< 0.001	91.6	− 0.22	0.828
Europe and America	3	0.01 (− 0.28 to 0.31)	0.06	0.970	0.0	5.41	0.116
Age							
Age ≥ 45	2	2.05 (0.06 to 4.04)	19.71	< 0.001	94.9	NA	NA
Age < 45	5	0.15 (− 0.64 to 0.93)	29.89	< 0.001	86.6	0.57	0.610
Combined	7	0.69 (− 0.23 to 1.62)	89.84	< 0.001	93.3	0.25	0.809

*N* number, *SMD* standard mean difference, *CI* confidence interval, *AML* acute myeloid leukemia, *VEGF* vascular endothelial growth factor

#### Sensitivity analyses and publication bias

**Fig. 3:**
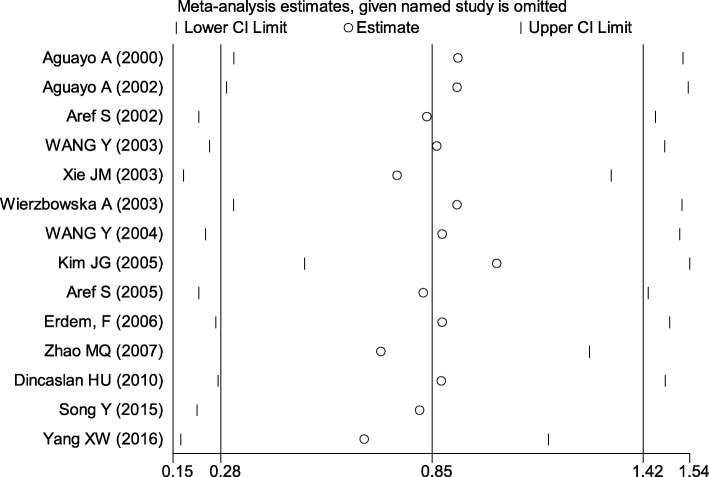
Sensitivity analyses by excluding one study at a time

**Fig. 4:**
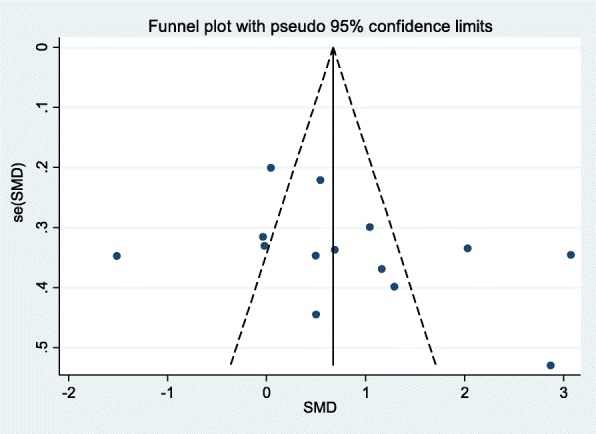
Funnel plot (with pseudo 95% confidence intervals) with the standard error of the VEGF difference plotted against the mean difference of VEGF of each study. SE: standard error

Sensitivity analyses showed no significant change when any one study was excluded using random-effects methods (*P* > 0.05) (Fig. 3). The asymmetry of the funnel plot was evaluated by the Egger’s test, while Egger’s linear regression test showed no publication bias (*P* > 0.05) (Fig. 4).

## Discussion

Acute myeloid leukemia (AML) is an aggressive and heterogeneous hematological disease that primarily affects older adults and is characterized by the expansion of immature myeloid blasts in the bone marrow [43]. Although leukemia research has been studied for a long time, the long-term survival of elderly patients with AML remains very low [44].

VEGF is an important regulator of physiological and pathological angiogenesis, which can promote endothelial cell proliferation and tumor growth, and the level of VEGF is associated with clinical outcome in hematologic malignancies including AML [13]. In AML patients, AML blasts produce and secrete VEGF, leading to elevated levels of VEGF in serum and bone marrow, indicating that VEGF plays an important role in AML as an autocrine growth factor [45].

The level of VEGF in AML patients remains controversial. Some studies showed different conclusions probably due to the limited sample sizes, making it difficult to get an objective and actual views. In order to solve this dispute and draw a more objective conclusion, we conducted a meta-analysis. It can increase the sample sizes by combining several independent research results, increase the credibility of the conclusion through comprehensive analysis, and solve the inconsistency of research results, so as to obtain a relatively objective result. To conclude, our meta-analysis showed the increased circulating level of VEGF in AML patients. Of the 649 AML patients included in the 14 studies, Aguayo et al. [16] included patients with relapse, while Dincaslan et al. [31] included one patient with relapse and one secondary AML patient, and the remaining 12 studies were all newly diagnosed AML patients. Our conclusion was consistent with a recent review, which indicated that the level of VEGF was elevated in AML patients at the time of diagnosis and at relapse [46]. A meta-analysis had already shown that patients with high levels of VEGF expression had worse event-free survival (EFS) and poorer overall survival (OS) [47]. In addition, the level of VEGF was decreased in AML patients after treatment or remission compared to healthy controls according to the review [46]. This may suggest that reducing the level of VEGF may allow the disease to progress to a better state, or even to a state of remission. VEGF and its receptors may provide promising targets in AML.

This meta-analysis mainly shows that the circulating levels of VEGF in AML patients was increased, suggesting that the high circulating levels of VEGF may serve as a biomarker in AML patients. The increased levels of VEGF may be used as a prognostic indicator to assess the severity of AML disease, providing new insights for future diagnosis, monitoring, and treatment of AML.

Heterogeneity was high in our systematic review and meta-analysis. First, our subgroup analysis showed that the sample size, sample type, region, and age were potential sources of significant heterogeneity. Second, large difference of sample size between AML patients and the control group may be responsible for the heterogeneity. The third point is that some of the data obtained approximately by conversion may lead to the heterogeneity. Next, one third of articles had no criteria for the classification of AML, which may contribute to the heterogeneity. Furthermore, among the 649 AML patients included in this study, different clinical characteristics such as different platelet and leukocyte counts, basic diseases, complications, and tumor load level may affect the level of VEGF, which may be the source of heterogeneity.

There are several limitations that should be noted in our meta-analysis. First of all, there are several articles with a small sample size that may affect our results, and the large gap in sample size between the patient group and the control group may affect the results and may increase heterogeneity. Second, we did not find the full text of five literatures, which may meet our inclusion and exclusion criteria. In addition, we are unable to obtain information from some unreported or unpublished studies. Next, some patients with AML have incomplete age, gender, and other basic characteristics and lack of sufficient data for subgroup analysis. For example, we only had seven studies with age data, one of which is inaccurate, so our subgroup analysis may not be accurate. Furthermore, some of the data obtained approximate figures by conversion, which might bias the results. Last but not least, the current study has not yet been registered online, and although we are still following the steps of systematic evaluation, there may still be small deviations.

Apart from these limitations, this meta-analysis also has its strengths and benefits. First, compared to individual studies, our meta-analysis enhanced generalizability by combining 14 studies from 6 countries. Second, subgroup analysis was performed to further explore potential sources of significant heterogeneity. Third, no publication bias was detected and sensitivity analysis was stable. Fourth, this is the first meta-analysis of VEGF levels in AML patients that provides a relatively reliable result compared to individual studies.

## Conclusions

In conclusion, patients with AML, mainly those newly diagnosed and untreated, had higher levels of VEGF than healthy controls. Furthermore, the level of VEGF in AML patients was correlated with sample size, sample type, region, and age. However, further analysis is still needed to determine the exact relationship between AML and VEGF. Basic data such as gender and age of AML patients need to be further improved to determine whether some basic characteristics of AML patients are sources of heterogeneity.

## Supplementary information

**Additional file 1:.** Preferred Reporting Items for Systematic Reviews and Meta-analysis (PRISMA) 2009 checklist.

**Additional file 2:.** Search strategy.

**Additional file 3:.** Specific calculations of our systematic reviews and meta-analysis.

**Additional file 4:.** The Risk Of Bias In Non-randomized Studies of Interventions (ROBINS-I) assessment tool.

## Data Availability

The datasets generated and/or analyzed during the current study are available from the corresponding author on reasonable request.

## Abbreviations

AML: Acute myeloid leukemia
VEGF: Vascular endothelial growth factor
PRISMA: Preferred Reporting Items for Systematic Reviews and Meta-analysis
NOS: Newcastle-Ottawa scale
ROBINS-I: Risk of Bias In Non-randomized Studies of Interventions
SMD: Standardized mean difference
ELISA: Enzyme-linked immunosorbent assay
95% CI: 95% confidence interval
