# Supplementary material for: Increased circulating vascular endothelial growth factor in acute myeloid leukemia patients: a systematic review and meta-analysis
Source: Syst Rev. 2020 May 6;9:103. doi: 10.1186/s13643-020-01368-9 (PMC7201671; doi:10.1186/s13643-020-01368-9)
Supplement: Supplementary file 2 — Additional file 2:. Search strategy. [file 13643_2020_1368_MOESM2_ESM.docx]

## Additional file 2 search strategy

| Databases | Date searched | No. retrieved |
| --- | --- | --- |
| PubMed | 04/02/2020 | 395 |
| Cochrane | 04/02/2020 | 16 |
| EMBASE | 04/02/2020 | 1343 |

1. PubMed- MESH

| 1. MESH: Vascular Endothelial Growth Factor A | 1. MESH: acute myeloid leukemia |
| --- | --- |
| Entry Terms:  •Vascular Endothelial Growth Factor-A  •VEGF-A  •Vasculotropin  •VEGF  •Vascular Endothelial Growth Factor  •Vascular Permeability Factor  •Permeability Factor, Vascular  •Glioma-Derived Vascular Endothelial Cell Growth Factor  •Glioma Derived Vascular Endothelial Cell Growth Factor  •GD-VEGF | Entry Terms:  •Acute Myeloid Leukemia  •Acute Myeloid Leukemias  •Leukemias, Acute Myeloid  •Myeloid Leukemias, Acute  •ANLL  •Leukemia, Acute Myelogenous  •Leukemia, Acute Myeloid  •Leukemia, Myeloblastic, Acute  •Leukemia, Myelocytic, Acute  •Leukemia, Myelogenous, Acute  •Leukemia, Nonlymphoblastic, Acute  •Leukemia, Nonlymphocytic, Acute  •Myeloblastic Leukemia, Acute  •Acute Myeloblastic Leukemia  •Acute Myeloblastic Leukemias  •Leukemia, Acute Myeloblastic  •Leukemias, Acute Myeloblastic  •Myeloblastic Leukemias, Acute  •Myelocytic Leukemia, Acute  •Acute Myelocytic Leukemia  •Acute Myelocytic Leukemias  •Leukemia, Acute Myelocytic  ......(et al) |

1. Search strategies: No method or language restrictions were applied, and studies from all countries were eligible. No pubication years restricted, and the search deadline was February 2020.
2. PubMed

| # | Search (all fields) | Results |
| --- | --- | --- |
| 1 | Vascular Endothelial Growth Factor | 93262 |
| 2 | Vasculotropin | 50539 |
| 3 | VEGF | 77637 |
| 4 | VEGF-A | 52138 |
| 5 | #1 OR #2 OR #3 OR #4 | 104418 |
| 6 | acute myeloid leukemia | 77201 |
| 7 | AML | 31513 |
| 8 | acute nonlymphocytic leukemia | 77931 |
| 9 | ANLL | 77836 |
| 10 | #6 OR #7 OR #8 OR #9 | 83309 |
| 11 | #5 AND #10 | 395 |

1. Cochrane

| # | Search (all text) | Results |
| --- | --- | --- |
| 1 | Vascular Endothelial Growth Factor | 3900 |
| 2 | Vasculotropin | 1253 |
| 3 | VEGF | 4281 |
| 4 | VEGF-A | 380 |
| 5 | #1 OR #2 OR #3 OR #4 | 6174 |
| 6 | acute myeloid leukemia | 4357 |
| 7 | AML | 3947 |
| 8 | acute nonlymphocytic leukemia | 170 |
| 9 | ANLL | 121 |
| 10 | #6 OR #7 OR #8 OR #9 | 5709 |
| 11 | #5 AND #10 | 16 |

1. EMBASE

| # | Search (all fields) | Results |
| --- | --- | --- |
| 1 | ‘Vascular Endothelial Growth Factor’ | 75456 |
| 2 | Vasculotropin | 149073 |
| 3 | VEGF | 98203 |
| 4 | VEGF-A | 9603 |
| 5 | #1 OR #2 OR #3 OR #4 | 165045 |
| 6 | ‘acute myeloid leukemia’ | 95287 |
| 7 | AML | 62485 |
| 8 | ‘acute nonlymphocytic leukemia’ | 1547 |
| 9 | ANLL | 1768 |
| 10 | #6 OR #7 OR #8 OR #9 | 113489 |
| 11 | #5 AND #10 | 1343 |

1. Included studies (14 articles)

[16]. Aguayo A, Kantarjian H, Manshouri T, Gidel C, Estey E, Thomas D, et al. Angiogenesis in acute and chronic leukemias and myelodysplastic syndromes. Blood 2000;96(6):2240-5. https://doi.org/ 10.1182/blood.V96.6.2240

[17]. Kim JG, Sohn SK, Kim DH, Baek JH, Lee NY, Suh JS, et al. Clinical implications of angiogenic factors in patients with acute or chronic leukemia: hepatocyte growth factor levels have prognostic impact, especially in patients with acute myeloid leukemia. Leuk Lymphoma 2005;46(6):885-91. https://doi.org/ 10.1080/10428190500054491

[20]. Yang XW, Ma LM, Zhao XQ, Ruan LH. [Clinical Curative Efficacy of Lenalidomide Combined with Chemotherapy for Acute Leukemia and Its Impact on VEGF]. Zhongguo Shi Yan Xue Ye Xue Za Zhi 2016;24(3):702-6. https://www.ncbi.nlm.nih.gov/pubmed/27342494?dopt=Abstract

[21]. Song Y, Tan Y, Liu L, Wang Q, Zhu J, Liu M. Levels of bone marrow microvessel density are crucial for evaluating the status of acute myeloid leukemia. Oncol Lett 2015;10(1):211-215. https://doi.org/ 10.3892/ol.2015.3209

[22]. Zhao MQ, Zhao HG. [Serum level of angiogenesis-related cytokins in patients with myelodysplastic syndrome]. Zhongguo Shi Yan Xue Ye Xue Za Zhi 2007;15(3):519-22. https://www.ncbi.nlm.nih.gov/pubmed/17605857?dopt=Abstract

[23]. Erdem F, Gündogdu M, Kiziltunç A. Serum vascular endothelial growth factor level in patients with hematological malignancies. European Journal of General Medicine 2006;3(3):116-120. http://www.bioline.org.br/abstract?gm06024

[24]. Xie JM, Qi ZH. [Clinical significance and expression of vascular endothelial growth factor in serum of patients with acute leukemia]. Hunan Yi Ke Da Xue Xue Bao 2003;28(2):183-5. https://www.ncbi.nlm.nih.gov/pubmed/12934374?dopt=Abstract

[25]Aref S, Mabed M, Sakrana M, Goda T, El-Sherbiny M. Soluble hepatocyte growth factor (sHGF) and vascular endothelial growth factor (sVEGF) in adult acute myeloid leukemia: relationship to disease characteristics. Hematology 2002;7(5):273-9. https://doi.org/ 10.1080/1024533021000037207

[26]. Aref S, El SM, Goda T, Fouda M, Al AH, Abdalla D. Soluble VEGF/sFLt1 ratio is an independent predictor of AML patient out come. Hematology 2005;10(2):131-4. https://doi.org/ 10.1080/10245330500065797

[27]. Aguayo A, Kantarjian HM, Estey EH, Giles FJ, Verstovsek S, Manshouri T, et al. Plasma vascular endothelial growth factor levels have prognostic significance in patients with acute myeloid leukemia but not in patients with myelodysplastic syndromes. Cancer 2002;95(9):1923-1930. https://doi.org/ 10.1002/cncr.10900

[28]. Wang Y, Xiao ZJ, Liu P, Yang C, Yang RC, Cai YL, et al. [Expression of vascular endothelial growth factor and its receptors KDR and Flt1 in acute myeloid leukemia]. Zhonghua Xue Ye Xue Za Zhi 2003;24(5):249-52. https://www.ncbi.nlm.nih.gov/pubmed/12859876?dopt=Abstract

[29]. Wang Y, Xiao ZJ, Liu P, Peng Z, Han ZC. [Expression of angiogenic factors and their clinical significances in acute myeloid leukemia]. Ai Zheng 2004;23(11 Suppl):1423-7. https://www.ncbi.nlm.nih.gov/pubmed/15566649?dopt=Abstract

[30]. Wierzbowska A, Robak T, Wrzesien-Kus A, Krawczynska A, Lech-Maranda E, Urbanska-Rys H. Circulating VEGF and its soluble receptors sVEGFR-1 and sVEGFR-2 in patients with acute leukemia. Eur Cytokine Netw 2003;14(3):149-53.

https://www.ncbi.nlm.nih.gov/pubmed/14656688?dopt=Abstract

[31]. Dincaslan HU, Yavuz G, Unal E, Tacyildiz N, Ikinciogullari A, Dogu F, et al. Does serum soluble vascular endothelial growth factor levels have different importance in pediatric acute leukemia and malignant lymphoma patients? Pediatr Hematol Oncol 2010;27(7):503-16. https://doi.org/ 10.3109/08880018.2010.493574
