# Supplementary material for: Increased circulating vascular endothelial growth factor in acute myeloid leukemia patients: a systematic review and meta-analysis
Source: Syst Rev. 2020 May 6;9:103. doi: 10.1186/s13643-020-01368-9 (PMC7201671; doi:10.1186/s13643-020-01368-9)
Supplement: Supplementary file 3 — Additional file 3:. Specific calculations of our systematic reviews and meta-analysis. [file 13643_2020_1368_MOESM3_ESM.docx]

1. We would use the formulas in the following three articles to calculate

①(Estimating the mean and variance from the median, range, and the size of a sample. doi:10.1186/1471-2288-5-13, This article is available from: <http://www.biomedcentral.com/1471-2288/5/13.> )

②(A Standard Error: Distinguishing Standard Deviation From Standard Error. DOI: 10.2337/db13-0692, PMID=23881207) ;

③(Bisphosphonates for periprosthetic bone loss after joint arthroplasty: a meta-analysis of 14 randomized controlled trials. Osteoporos Int (2012) 23:1823–1834; DOI 10.1007/s00198-011-1797-5. )

1. We would like to show the detailed process of calculations:

**Aguayo A et al(2000)**

1. **AML, n=115,**

**VEGF,** median=30.43, range(21.47-439.25), n>25,mean=median=30.43

N>70, s=(439.25-21.47)/6=69.63

VEGF(mean±sd ): 30.43±69.63

1. **control,n=11,**

**VEGF:** median=26.74, range(23.17-53.88),

n≤25, mean=(23.17+2*26.74+53.88)/4=32.63

n≤15，

S^2^=[(23.17-2*26.74+53.88)*(23.17-2*26.74+53.88)/4+(53.88-23.17)*(53.88-23.17)]/12=90.166

S=9.50

VEGF(mean±sd ):32.63±9.50

**Aguayo A et al(2002)**

1. **AML, n=58,**

**VEGF,** median=30.63, range(21.47-389.82)

N>25, mean=median=30.63

15<n≤70, s=(389.82-21.47)/4=92.09

VEGF(mean±sd ):30.63±92.09

1. **Control, n=43**

**Age:** median=39, range(24-79), N>25, mean=median=39, s=(79-24)/4=13.75 age(mean±sd) =39±13.75

**VEGF:** median=27.3, range(23.17-91.5), mean=median=27.3, s=(91.5-23.17)/4=17.08; VEGF(mean±sd ): 27.3±17.08

**Aref S et al(2002)**

1. **AML,n=63,**

**Age:** median=47, range(20-70)

N>25, mean=47, S=(70-20)/4=12.5

Age(mean±sd ):47.00±12.50

**VEGF**: median=78, range(32-221)

N>25, mean=78,s=(221-32)/4=47.25

VEGF(mean±sd ):78.00±47.25

1. **Control, n=15**

**VEGF:**median=27, range(16-62.1)

n≤25，mean=(16+2*27+62.1)/4=33.03,

n≤15，S2=[（16-2*27+62.1）*（16-2*27+62.1）/4 +（62.1-16）*（62.1-16）]/12=189.201

S=13.76

VEGF(mean±sd ):33.03±13.76

**Wang Y et al (2003)**

**AML**，n=39，

**Age:** median=42, range(14-73), n>25, mean=median=42,

15<n≤70, s=(73-14)/4=14.75,age(mean±sd) =42±14.75

**VEGF**：can get directly from the article

**Wierzbowska A et al( 2003)**

VEGF was measurable in 38 AML patients and 12 controls.

**(1)AML ,n=38,**

**VEGF,** median=32.6, range (0-2604.8), n>25, mean=median=32.6,

15<n≤70, s=(2604.8-0)/4=651.2,VEGF(mean±sd)=32.6±651.2

**(2)control, n=12,**

**VEGF: median=34.9, range(0-107.8), n**≤25, mean=(0+2*34.9+107.8)/4=44.4

n≤15，S^2^=[(0-2*34.9+107.8)*(0-2*34.9+107.8)/4+107.8*107.8]/12=998.48666

S=31.60

VEGF(mean±sd)=44.4±31.60

**Wang Y et al (2004)**

**AML,** n=107

**Age,** median=42, range(12-83), n>25, mean=median=42, n>70, s=(83-12)/6=11.83

age(mean±sd)=42±11.83

VEGF can get directly from the article.

**Kim JG et al(2005)**

1. **AML, n=28,**

**Age ,**n=30,median=41.5,range(15-74), n>25, mean=median=41.5,

15<n≤70, s=(74-15)/4=14.75, age(mean±sd) =41.5±14.75

**VEGF:** median=54.3, range(2.8-455.4), n>25, mean=median=54.3

15<n≤70, s=(455.4-2.8)/4=113.15; VEGF(mean±sd)=54.3±113.15

1. **control, n=17,**

**VEGF :**median=202.9, range(2.5-547.5)

n≤25,mean=(2.5+2*202.9+547.5)/4=238.95

15<n≤70, s=(547.5-2.5)/4=136.25

VEGF(mean±sd)=238.95±136.25

**Dincaslan et al(2010)**

1. AML, n=7,

**VEGF:** median=52, range(2-1040);

n≤25，mean=(2+2*52+1040)/4=286.5,

n≤15，S^2^=[(2-2*52+1040)*(2-2*52+1040)/4+(1040-2)*(1040-2)]/12=108117.08333333

S=328.81

(2)control, n=20, median=116, range(30-500)

n≤25, mean=(30+2*116+500)/4=190.5，

15<n≤70, s=(500-30)/4=117.5

Erdem F et al (2006) do not need to calculate, we can get the value directly from the article.

Zhao MQ et al (2007) do not need to calculate, we can get the value directly from the article.

Song y et al(2015) do not need to calculate, we can get the value directly from the article.

Xie JM et al(2003) do not need to calculate, we can get the value directly from the article.

**Yang XW et al(2016):** about the translated values of this article, we would go into details.

Table 2 was from Yang XW et al(2016), and all data was showed in mean±SE in this article.

The total number of newly diagnosed AML patients were divided into two groups. The two groups would accept two different therapies. One was CP (chemotherapy + placebo), and the other was LC(lenalidomide + chemotherapy). What we need was the data of newly diagnosed (BT: before treatment) AML patients. Since we can only get separate data, we need to merge the data.

(AML) CPBT: n1=35, mean1±SE1=275.5±7.658

(AML) LCBT: n2=33, mean2±SE2=312.0±11.07


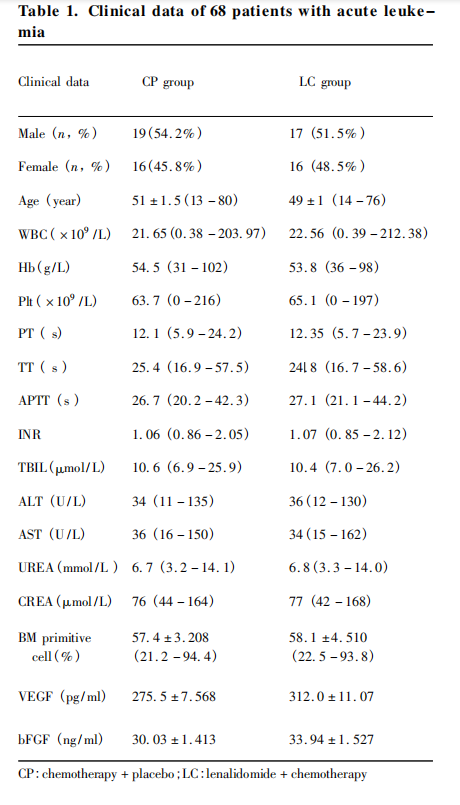


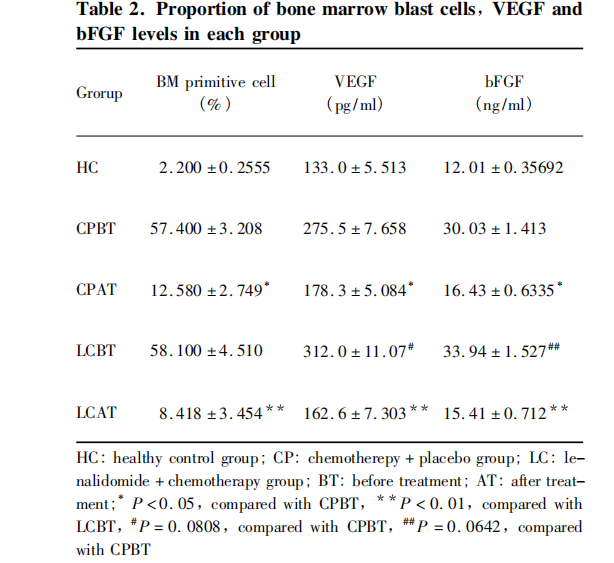


We used the following formula to calculate the value, and we would cite the following article in our revised manuscript.

(1)SE, standard error; SD,standard deviation, SE=SD/√n; A Standard Error: Distinguishing Standard Deviation From Standard Error. DOI: 10.2337/db13-0692, PMID=23881207

(2)Bisphosphonates for periprosthetic bone loss after joint arthroplasty: a meta-analysis of 14 randomized controlled trials. Osteoporos Int (2012) 23:1823–1834; DOI 10.1007/s00198-011-1797-5.


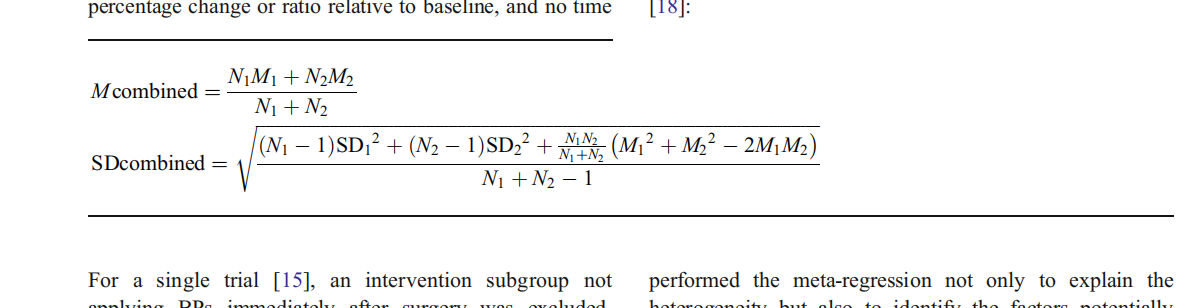


① First, we turned mean±SE to mean±SD by using the formula **SE=SD/√n;**

CPBT: N1, M1, S1(35, 275.5, 45.31)；

LCBT:N2, M2, S2(33, 312.0, 63.59)

② Second, we used the (2) formula to calculate the M combined, and the SD combined.

At last, we got the AML , n=68, VEGF (mean±sd)=293.21±57.54
